# Recent Advances in Erinacine A: Preparation, Biological Activities, and Biosynthetic Pathway

**DOI:** 10.3390/molecules31020219

**Published:** 2026-01-08

**Authors:** Jingyuan Wang, Huan Liu, Chunlei Wang, Chengwei Liu

**Affiliations:** 1 Key Laboratory of National Forestry and Grassland Administration on Chinese Herbal Medicine, College of Life Science, Northeast Forestry University, Harbin 150040, China; jingyuanwang4272@163.com (J.W.); 13193827058@163.com (H.L.); 2 State Key Laboratory of Utilization of Woody Oil Resource, Northeast Forestry University, Harbin 150040, China

**Keywords:** erinacine A, *Hericium erinaceus*, biosynthesis, biological activity, neuroprotection

## Abstract

Erinacine A, a cyathane diterpenoid derived from the medicinal and edible fungus *Hericium erinaceus*, is increasingly recognized for its potent neurotrophic and neuroprotective properties. It demonstrates significant therapeutic promise for neurodegenerative disorders, such as Alzheimer’s and Parkinson’s disease, primarily by stimulating the synthesis of nerve growth factor (NGF). However, the clinical applicability of erinacine A is currently restricted by its low yield from natural sources and high production costs. This challenge has spurred significant research focused on optimizing its production. This review provides a comprehensive overview of the current advancements in the fermentation-based preparation of erinacine A, including both liquid and solid-state cultivation techniques. Furthermore, we summarize its diverse biological activities, spanning neuroprotection, anticancer, and anti-inflammatory effects, and detail the recent discoveries elucidating its complex biosynthetic pathway. This consolidated overview offers insights into strategies for enhancing its production and supports its ongoing development as a therapeutic agent.

## 1. Introduction

For millennia, mushrooms have been an integral component of traditional medicine, valued for their extensive therapeutic properties. Among these medicinal fungi, *Hericium erinaceus* (commonly known as Lion’s Mane or Yamabushitake) is renowned for its exceptional culinary and medicinal qualities [1]. The taxonomy of *H. erinaceus* follows Basidiomycota, Agricomycotina, Agaricomycetes, Incertaesedis, Russulales, and Hericiaceae. *Hericium erinaceus* is predominantly a saprophyte, but occasionally it may also be a weak parasite of trees. *H. erinaceus* primarily inhabits Fagus and Quercus trees, specifically those of *Q. cerris*, *frainetto*, and *gussonei*. The fungus exhibits a preference for *Aesculus hippocastanus*, *Albizia julibrissin*, *Alnus glutinosa*, *A. incana*, *Carpinus betulus*, *Populus tremula*, and *Tilia cordata*, although its occurrence on these host plants is infrequent [2,3]. Its native range spans North America, Europe, and Asia.

Large-scale cultivation of *H. erinaceus* is economically viable, as it can utilize low-cost agricultural waste, such as straw and wood chips, as substrates. Globally, its commercial cultivation has become increasingly popular, especially in Asia and the US [4]. It is frequently included in diets throughout East Asian nations due to its exceptional health benefits [5]. *H. erinaceus* has a long history of use in traditional medicine, particularly within the frameworks of traditional healing practices. Traditionally used in Chinese medicine, it is recognized for its potential to prevent and treat oxidative stress-related disorders [6,7]. Modern scientific inquiry has begun to unravel the mechanisms behind these traditional uses, with studies suggesting that *H. erinaceus* may indeed stimulate nerve regeneration and offer protection against neurodegenerative diseases. Furthermore, its application in supporting digestive health is well documented, as it was traditionally used to treat conditions such as gastritis and ulcers, attributed to its protective effects on the gastric mucosa [8,9,10]. Furthermore, extensive research has demonstrated that *H. erinaceus* possesses a broad spectrum of biological activities, including antioxidant [11], anti-inflammatory [12], hypolipidemic [13], anticoagulant [14], antibacterial [15], hypoglycemic and antidiabetic [16,17,18], as well as anticancer properties [19,20].

This therapeutic potential is largely attributed to erinacines, a class of bioactive cyathane diterpenoid chemicals that promote the synthesis of nerve growth factor (NGF) [21,22]. Research indicates that these substances present in mycelium exhibit potent neuroprotective properties. For example, erinacines A and S can decrease amyloid β deposition and increase the production of insulin-degrading enzyme (IDE), whereas erinacines A-I stimulate the release of NGF [23]. Moreover, erinacine E has been found to modulate neuropathic pain. The remaining erinacines either have different pharmacological actions or are still in the discovery stage. Erinacines can cross the blood–brain barrier [24,25]. Among these compounds, Erinacine A, an active compound isolated from cultured *H. erinaceus* mycelium, stands as the most extensively studied component. The absolute bioavailability of erinacine A was estimated to be 24.39%, higher than the 15.13% of erinacine S in the previous study [26,27]. It exhibits numerous biological characteristics, including antioxidant, anti-aging, immunomodulatory, anticancer, and neuroprotective activities. This may bring scientists one step closer to creating more effective medicines for neurodegenerative diseases.

However, despite its immense therapeutic promise, the clinical applicability of erinacine A is severely restricted by its high cost and low yield. Consequently, research on erinacine A continues to garner significant interest. In order to offer insights into its production, study, and therapeutic application, this review describes and investigates the recent advances in the fermentation-based preparation, biological activities, and biosynthesis of erinacine A.

## 2. Fermentation Preparation of Erinacine A

**Figure 1:**
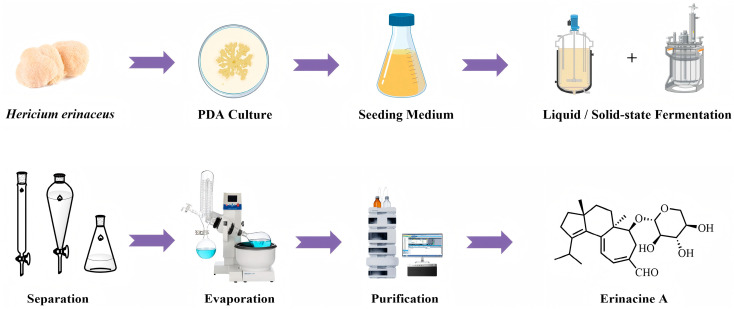
Fermentation preparation of erinacine A.

The primary natural source of erinacines is the mycelium of *H. erinaceus*. As roughly shown in Figure 1, the main methods for obtaining this compound involve cultivating the mycelium using solid-state and submerged liquid fermentation, followed by extraction and separation. Due to the low natural abundance of erinacines, optimizing the cultivation procedure of *H. erinaceus* mycelium to increase their yield is essential.

Krzyczkowski et al. [28] optimized the liquid fermentation medium for *H. erinaceus* with a particular medium formulation (containing 69.87 g/L glucose, 11.17 g/L casein peptone, 1.45 g/L sodium chloride, 55.24 mg/L zinc sulfate, potassium dihydrogen phosphate 1.0 g/L, and pH adjusted to 4.5). After 8 days of cultivation in a bioreactor, the yield of erinacine A reached 192 ± 42 mg/L. After 14 days in a 100 L bioreactor, the growth medium (0.5% yeast extract, 4% glucose, 0.5% soybean meal, 0.25% peptone, 1% oats, 0.05% KH_2_PO_4_, pH 5) produced 206 ± 7 mg/L (17.34 mg/g) of erinacine A [23]. Another useful strategy for increasing the yield of erinacine chemicals during the liquid fermentation of *H. erinaceus* is to supplement the system with additional substances like metal ions, and trace elements. For instance, Chang et al. [29] added extra metal ions, including ferrous iron, copper, manganese, and nickel, to the *H. erinaceus* cultivation system. Mycelium biomass increased to 16.8 g/L as a result, and erinacine A production similarly reached (225 ± 54) mg/L. There have also been reports of investigations on the use of large-scale bioreactors for the synthesis of erinacine A. A 20-ton fermenter with a medium that contained 2.5 g/L yeast extract, 45 g/L glucose, 5 g/L soybean meal, 2.5 g/L peptone, and 0.5 g/L magnesium sulfate was used in tests by Li et al. [30]. Erinacine A was produced at 5 mg/g dry mycelium weight after 12 days of fermentation at pH 4.5.

In addition to liquid fermentation, the erinacine A content can be improved by adding inorganic materials or trace elements to solid fermentation media in addition to liquid fermentation technology. According to research by Gregori et al. [31], the best promoting effect on erinacines synthesis happens at a sodium chloride content of 0.56% and a casein content of 3.4% when trace elements are added to solid fermentation media (substrate was based on a mixture of husked and paddy millet in ratio = 7:2, with 50% water content) at various doses. According to Cheng et al. [32], adding zinc sulfate or sodium chloride to the growth medium (polished rice, corn kernel, adlay kernel, brown rice, red bean, and sesame) greatly enhanced the production of erinacine A, reaching 165.36 mg/g and 120.97 mg/g, respectively. The yield of erinacine A is directly impacted by the choice of components and their proportionate formulation in the culture medium. Enhancing erinacine A production also requires adding the right concentrations of hormones, trace minerals, and other compounds to the culture environment.

Additionally, after the first isolation and identification of erinacine A from the mycelium [33], a number of modifications for erinacine A extraction and separation have been carried out by researchers. In order to separate the target compound erinacine A from *H. erinaceus* mycelium, Valu et al. [34] used ultrasonic extraction technology. They also introduced response surface methodology to optimize important extraction parameters, including solid-to-liquid ratio and extraction duration, which affect extraction efficiency. Experimental results show that when the extraction process lasts 45 min and the solvent-to-material ratio is maintained at 20 mL/g, the extraction yield of erinacine A can reach 17.50%. Furthermore, Liu et al. [35] effectively separated erinacine A with 95% purity using high-speed countercurrent chromatography (HSCCC) and a two-phase solvent mixture of n-hexane/ethyl acetate/methanol/water (volume ratio 4.5:5:4.5:5). Significant differences in the amount of erinacine A across several fungal strains were also noted. Naumoska et al. [36] combined normal-phase (NP) and reversed-phase (RP) chromatography to perform a two-dimensional fractionation. By choosing the best extraction solvent, the isolation process can be optimized, and chromatographic technique optimization can improve the final isolate’s purity. This production platform yielded 19.4 mg of erinacine A from approximately 130 g of mushroom material, with a chromatographic purity of 97.4%.

In conclusion, liquid fermentation offers advantages such as short production cycles, high efficiency, stable product quality, and convenient product extraction. However, it involves high equipment costs and susceptibility to contamination. Solid-state fermentation, on the other hand, features simpler equipment and lower costs but requires longer growth cycles compared to liquid fermentation. The ratio and makeup of the components of the culture medium play a major role in controlling the erinacine A yield. Its synthesis efficiency can be greatly increased by adding hormones and trace elements in the right amounts. It is currently challenging to satisfy industrial production standards since erinacine A separation and purification technology is limited to laboratory scale. In order to overcome technological obstacles and eventually achieve large-scale, reliable production of erinacine A, future efforts should concentrate on process scale-up and systematic optimization along the entire chain, from strain cultivation to extraction and isolation of active components.

## 3. The Biological Activity of Erinacine A

**Figure 2:**
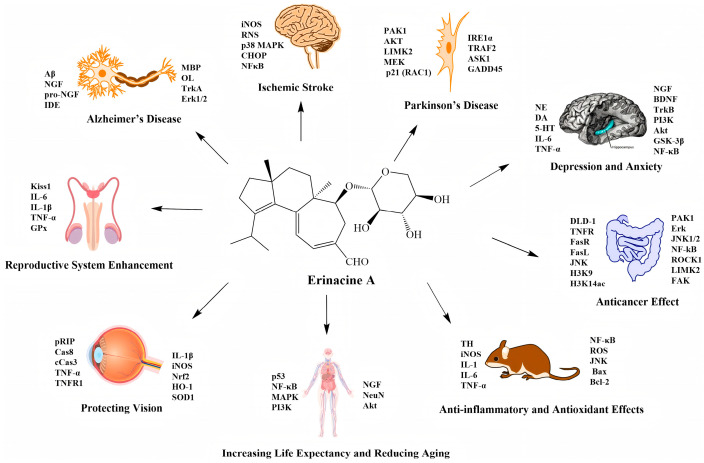
The biological activity of erinacine A.

Recent pharmacological studies have identified erinacine A as one of the primary compounds responsible for the health benefits of *H. erinaceus*. As shown in Figure 2, its biological activities cover a wide range of important health areas, from neuroprotection to anticancer effects and from inflammation management to aging delay, providing innovative natural remedies for common chronic illnesses and health issues. Its method of action includes both comprehensive augmentation of basic physiological processes like antioxidant and anti-inflammatory responses and targeted control of certain signaling pathways.

### 3.1. Protection Against Alzheimer’s Disease

Neurofibrillary tangles, β-amyloid (Aβ) plaque development, and loss of neurons and synapses are the hallmarks of Alzheimer’s disease (AD), an age-related progressive neurodegenerative illness [37]. These pathologies are linked to impaired neurogenesis and NGF deficiency, which contribute to the disease’s advancement [38,39,40,41,42,43].

Tsai et al. [44] administered oral doses of *H. erinaceus* mycelium (containing 3 mg/mL erinacine A) to 5-month-old APPswe/PS1dE9 transgenic mice for 30 days. The findings showed that this fungus suppressed the recruitment and activation of plaque-associated microglia and astrocytes in addition to lowering the brain β-amyloid (Aβ) plaque burden in rats. but also raised the expression of insulin-degrading enzyme (IDE), the ratio of NGF to proNGF, the number of newly created neurons in the dentate gyrus region, and the proliferation of neuronal cells. Bui et al. [45] employed a novel primary mixed glial cell model and advanced bioinformatics tools to administer erinacines at a dose of 30 mg/kg/day to HFSTZ-APP/PS1 mice. The results demonstrated that erinacines reduced the activation of plaque-associated microglia and astrocytes. This effect may further alleviate the inflammatory environment in the brain, thereby enhancing neurogenesis. Similarly, Tzeng et al. [46] found that both erinacine A and S compounds inhibited Aβ plaque growth, reduced neuroinflammation, and increased insulin-degrading enzyme (IDE) levels; only erinacine A decreased levels of insoluble β-amyloid (Aβ) and the C-terminal fragment (CTF) of amyloid precursor protein (APP) and reduced Aβ production.

Erinacine A promotes oligodendrocyte maturation, enhances neuronal survival, and stimulates neurite outgrowth. Huang et al. [47] found that treating ex vivo cerebellar slices from Sprague-Dawley (SD) rats with erinacine A (0.1, 1 ng/mL) and erinacine S (0.1 ng/mL) more effectively stimulated myelin basic protein (MBP) expression on neuronal fibers within oligodendrocytes (OL) and increased OL numbers. The decline of neurotrophins (NTs) is an important factor in the pathogenesis of neurodegenerative diseases (ND) [48]. In fact, genetic or direct induction of NGF has already been elucidated as a promising treatment for ND [49,50]. Zhang et al. [51] found that erinacine A induced neuritogenesis in PC12, acting as a stimulus for NGF potency instead of NGF level. Furthermore, analysis of cellular signaling pathways revealed that NGF-induced neurite outgrowth potentiated by erinacine A is completely TrkA-mediated and partially Erk1/2-dependent.

In a human study, Li et al. [52] conducted a pilot double-blind placebo-controlled study on patients with mild AD. The group receiving erinacine A-containing *H. erinaceus* mycelium extract (EAHE) showed enhanced cognitive effects, which was attributed to the stimulation of NGF synthesis and neurogenesis.

### 3.2. Protection Against Ischemic Stroke

Stroke is a leading cause of disability and the second leading cause of death worldwide. As the global population aged 65 and older grows faster than any other age group, the incidence of stroke is also increasing [53]. Ischemic stroke, the most common type of stroke, is caused by blood vessel occlusion, reducing blood supply to a specific area of the brain, leading to oxidative stress, inflammation, and neuronal cell death [54,55,56,57].

Lee et al. [58] demonstrated that oral administration of *H. erinaceus* mycelium rich in erinacine A (50 and 300 mg/kg) reduced total cerebral infarct volume in a rat model of focal cerebral ischemia. This protection was associated with reduced levels of inflammatory cytokines (IL-1β, IL-6, TNF-α) and suppression of reactive nitrogen species by downregulating iNOS, p38 MAPK, and CHOP. Allen et al. [59] found that rats treated with *H. erinaceus* mycelium rich in erinacine A exhibited increased neuronal survival following brain injury. The proposed mechanism of action suggests that *H. erinaceus* mycelium, rich in erinacine A, may reduce neuronal apoptosis and decrease stroke probability in rat brains by targeting iNOS reactive nitrogen species (RNS) and the p38 mitogen-activated protein kinase (MAPK)/CCAAT-enhancer-binding protein homolog (CHOP) pathway. Hsu et al. [60] used both in vitro (OGD) and in vivo (tHI) models to show that erinacine A mitigates neuronal and astrocyte damage. The protective effect was attributed to the inhibition of the NFκB signaling pathway, which is associated with pro-inflammation and astrocyte proliferation.

### 3.3. Protection Against Parkinson’s Disease

One of the most prevalent adult-onset movement disorders in the world is Parkinson’s disease (PD) [61]. It is brought on by a variety of hereditary and environmental variables that cause progressive dopaminergic neurodegeneration and death in the striatum and substantia nigra. The MPTP-induced dopamine neuron damage model is widely used in the development of PD intervention drugs [62].

Lee et al. [63] found that post-treatment with erinacine A (30 mg/kg) or *H. erinaceus* mycelium reduced motor dysfunction in MPTP-induced PD models. Experiments demonstrate that erinacine A treatment reduces MPTP-induced neurotoxicity by activating survival pathways involving PAK1, AKT, LIMK2, MEK, and Cofilin, while simultaneously suppressing cell death pathways including IRE1α, TRAF2, ASK1, GADD45, and p21 in N2a neurons and MPTP-induced Parkinson’s disease models.

### 3.4. Protection Against Depression and Anxiety

Depression is the most common psychiatric comorbidity, with prevalence rates as high as 87%, 75%, and 79% at any given time among patients with Alzheimer’s disease, Parkinson’s disease, and stroke, respectively [64]. Previous data indicate that patients with major depressive disorder exhibit significantly lower levels of NGF compared to healthy subjects [65]. Therefore, the mycelium of *H. erinaceus*, rich in erinacines, participates in the production of neurotrophic factors and is believed to play a role in depression.

Chronic restraint stress causes depression-like behavior and decreased hippocampus BDNF production in animal models [66]. The effects of erinacine A-enriched *H. erinaceus* mycelium on animals undergoing repeated chronic stress were thus examined by Chiu et al. [67]. HE administration (200 or 400 mg/kg) inverted the stress-induced decrease in neurotransmitters such as norepinephrine (NE), dopamine (DA), and serotonin (5-HT), as well as increases in inflammatory cytokines including interleukin-6 and tumor necrosis factor-α. The mechanism was linked to the activation of the BDNF/TrkB/PI3K/Akt/GSK-3β pathway and blockade of NF-κB signals. Li TJ et al. [68] administered two doses of *H. erinaceus* mycelium (75 mg/kg and 150 mg/kg) orally to C57BL/6 mice 20 min prior to the tail suspension test (TST), followed by a light exposure period. Results indicate that a 150 mg/kg dose of *H. erinaceus* mycelium improves anxiety in rodents (*p* < 0.05) and reverses TST-induced dark-phase non-rapid eye movement (NREM) sleep disturbances, confirming that higher intake (150 mg/kg) of *H. erinaceus* mycelium significantly ameliorates anxiety levels.

### 3.5. Anticancer Effect

According to a report by the World Health Organization (WHO), cancer is the leading cause of death globally [69], with breast, lung, and colorectal cancer having the highest incidence and mortality rates [70]. Although chemotherapy plays a pivotal role in cancer treatment, its side effects and the development of drug resistance have created an urgent need to find safer alternatives [71]. Erinacine A has been reported to exhibit significant neuroprotective and anticancer effects.

Lee et al. [72] found that erinacine A induces apoptosis in colorectal cancer cells (DLD-1) by activating both extrinsic and intrinsic pathways. This involved the upregulation of death receptors (TNFR, FasR, FasL) through histone (H3K9, H3K14ac) and changes in the JNK/p300/p50 signaling pathway. In other studies, Lee et al. [73] reported that erinacine A induced cytotoxicity in colorectal cancer cells (HCT-116 and DLD-1) via a surge in ROS production, inhibiting proliferation and invasion by targeting the ROCK1/LIMK2/Cofilin and PI3K/mTOR/p70S6K pathways. Kuo et al. [74] found that erinacine A induces apoptosis in the TSGH 9201 cell line, which is associated with sustained phosphorylation of PAK1 and the FAK/AKT/p70S6K pathway. The activation of these signaling pathways is accompanied by differential expression of 14-3-3 σ protein (14-3-3-σ) and microtubule-associated tumor suppressor candidate 2 (MTUS2), which erinacine A can utilize to limit the metastatic activity of gastric cancer cells. Lu et al. [75] confirmed in vivo that erinacine A inhibited DLD-1 tumor growth in mice, associated with increased NF-κB binding activities and decreased cell proliferation. Furthermore, Huang et al. [76] demonstrated that erinacine A could inhibit osteoclast generation and migration induced by breast cancer, suggesting potential in preventing bone metastasis by reducing TGF-β and MMP-9 production via Erk or JNK pathways.

### 3.6. Anti-Inflammatory and Antioxidant Effects

Erinacine A not only enhances resistance to oxidative stress but also suppresses inflammatory responses [77]. Hsu et al. [78] found that micronized *H. erinaceus* mycelium (HEM) rich in erinacine A protected against MPTP-induced damage by restoring dopamine levels and reducing oxidative stress markers (MDA, carbonyls). This was accompanied by an elevation in antioxidant enzyme activities (SOD, catalase, G6PDH, GRd).

Lee et al. [79] showed that erinacine A (5 mg/kg) or *H. erinaceus* mycelium (1 g/kg) pretreatment ameliorated LPS-induced neuroinflammation in rats. It prevented the expression of iNOS, TNF-α, and IL-1β in vivo and protected dopaminergic neurons by suppressing JNK and NF-κB phosphorylation. Chang et al. [29] found that HA effectively suppressed the ratio of Bax/Bcl-2 and caspase-3, resulting in promising inhibition of PC-12 cell apoptosis induced by Glu-insult. In vivo, it also led to reduced ROS levels and inflammatory cytokine production.

### 3.7. Increasing Life Expectancy and Reducing Aging

Dietary supplements containing natural products are increasingly studied for their potential to extend lifespan and mitigate age-related diseases. Erinacine A-enriched mycelium has shown promise in this area.

Wu et al. [80] utilized the ELAV-SCA3tr-Q78 fruit fly model to evaluate that *H. erinaceus* mycelium exerts anti-apoptotic effects by modulating the transcription factors p53 and NF-κB and their downstream events in SCA3 cells and Drosophila models disrupted by oxidative stress, thereby extending fly lifespan and improving motor activity. Wu et al. [81] demonstrated that treating ELAV-SCA3tr-Q78 transgenic fruit flies with ethanol extracts (HEME) of *H. erinaceus* mycelium containing 0.5% and 1% erinacine A extended their lifespan and improved motor activity. Li et al. [82] found that erinacine A-enriched *H. erinaceus* mycelium significantly extended the maximum, average, and median lifespan in a dose-dependent manner. This was potentially related to the regulation of oxidative stress pathways (MAPK, PI3K/Akt). Lee et al. [83] demonstrated in similar experiments that erinacine A-enriched *H. erinaceus* improved learning and memory in mouse brains and delayed degenerative aging.

Tsai et al. [84] found that in mice treated with *H. erinaceus* mycelium (1 g/kg) and erinacine A (43 mg/kg), reduced messenger RNA (mRNA) expression of tumor necrosis factor-α and interleukin-1β, and *H. erinaceus* mycelium-treated mice exhibited increased mRNA expression of NGF and NeuN in the hippocampus. Moreover, *H. erinaceus* mycelium and erinacine A also decreased body weight, abdominal fat, plasma glucose, serum and liver total cholesterol, and liver triacylglycerol. Thus, *H. erinaceus* mycelium may be a potential health-promoting supplement for minimizing the progression of aging and obesity-induced neurodegeneration by reducing metabolic abnormalities and neuroinflammatory cytokines and increasing neurogenesis factors. Ratto et al. [85] also demonstrated that *H. erinaceus* supplementation reversed age-related recognition memory decline and supported neurogenesis in frail mice.

### 3.8. Other Functions

Erinacine A has demonstrated protective effects in other systems. Hsu et al. [86] found that erinacine A exerted neuroprotective effects in a rat model of traumatic optic neuropathy. Results showed decreased levels of pRIP, Cas8, cCas3, TNF-α, TNFR1, IL-1β, and iNOS, along with increased levels of Nrf2, HO-1, and SOD1 in retinal samples from both erinacine A groups. This indicates that erinacine A exerts neuroprotective effects in experimental models of traumatic optic neuropathy by suppressing apoptosis, neuroinflammation, and oxidative stress, thereby preserving retinal ganglion cell (RGC) survival and maintaining visual function.

Additionally, Huang et al. [87] provided the first in vivo evidence that erinacine A-enriched *H. erinaceus* mycelium protected against male reproductive dysfunction induced by polystyrene microplastics. The treatment improved sperm count and quality and enhanced antioxidant activity (glutathione peroxidase). Additionally, HE treatment significantly increased Kiss1 concentration, upregulated follicle-stimulating hormone and testosterone levels, reduced the area of the seminiferous tubule lumen, and prevented a reduction in epithelial thickness.

In summary, erinacine A exhibits strong neurotrophic and neuroprotective effects. It provides prospective treatment opportunities for neurodegenerative illnesses, including Alzheimer’s and Parkinson’s, by encouraging neuronal repair and regeneration and modifying pertinent signaling pathways. Additionally, this chemical has potential uses in anti-aging and anticancer therapy. But the exact processes that underlie erinacine A’s function in protecting and regenerating neurons are still not fully understood. Most related studies have remained at the cellular or animal level, with few conducting preclinical or clinical research. There is a lack of human trial data, and in the majority of studies involving mice, the experimental dosage range for erinacine A lacks precise data. Many experiments utilize mycelium containing erinacine A rather than pure erinacine A. Moreover, there is not enough extensive clinical evidence to support its safety and effectiveness. The practical use of erinacine A is limited by these reasons. To create a strong theoretical basis for future clinical translation, further investigation into its particular processes in brain regeneration, neuroprotection, and anticancer actions is necessary. The effects and dosage of erinacine A on the human body also require further research.

## 4. Toxicology Studies

To date, all experimental studies have suggested that *H. erinaceus* mycelium is safe. Though not often observed, some side effects of HE supplementation include headaches [88], stomach discomfort, diarrhea [89], and epimenorrhea [90]. Importantly, comprehensive genotoxicity studies have found *H. erinaceus* mycelium to be non-mutagenic. It did not show adverse effects in the bacterial reverse mutation test (Ames test), the in vitro chromosome aberration test, or the in vitro erythrocyte micronucleus test, either with or without metabolic activation [91].

Preclinical in vivo studies in rat models further substantiate its safety profile. *H. erinaceus* powder demonstrated no toxicity in either acute or 90-day repeated-dose oral toxicity studies, and no effects were observed following a single oral dose of 2000 mg/kg body weight (bw). Additionally, powders were non-genotoxic in the bacterial reverse mutation assay (up to 5000 µg/plate) and in the in vivo mouse micronucleus assay (up to 2000 mg/kg bw) [92]. Acute toxicity studies showed that a single high dose of erinacine A-enriched *H. erinaceus* mycelium (EAHE) (5000 mg/kg body weight) did not result in mortality or treatment-related toxicity [93]. Sub-chronic and repeated-dose studies also found no adverse effects. Rats fed daily doses up to 3 g/kg showed no issues [30]. Similarly, a 13-week study with doses up to 2625 mg/kg found no significant toxicological effects, with no changes in physiological, hematological, or biochemical parameters [94]. Furthermore, developmental toxicity studies in pregnant rats (up to 2625 mg/kg) showed no differences in fetal weight, morphology, or implantation rates compared to controls, indicating the mycelium is non-teratogenic and safe during gestation at the tested doses [93]. Collectively, these data indicate that EAHE is virtually non-toxic and support its safe application in food preparation.

## 5. Biosynthesis of Erinacine A

Cyathane diterpenoids, represented by erinacines, constitute one of the most abundant classes of diterpenoid compounds produced by basidiomycetes [95]. In fungi, the synthesis of these compounds follows a common pattern: it begins with the mevalonate pathway (MVA) to generate the fundamental precursors isopentenyl pyrophosphate (IPP) and dimethylallyl pyrophosphate (DMAPP). Subsequently, isoprenyl transferase catalyzes the polymerization of these precursor units to form polyisoprene pyrophosphate with varying chain lengths. These linear molecules then undergo a series of complex reactions, including dephosphorylation, cyclization, and hydroxylation catalyzed by terpene synthase, ultimately constructing the diverse core carbon skeletons of terpenoid compounds. Ultimately, these skeletal structures undergo intricate refinement by various modifying enzymes, such as cytochromes P450, dehydrogenases, and transferases, to form end products that exhibit vastly different structures and biological functions. It is noteworthy that the downstream modification pathways of terpenoid compounds exhibit a high degree of species specificity, which is precisely the root cause of their structural and functional diversity [96].

**Figure 3:**
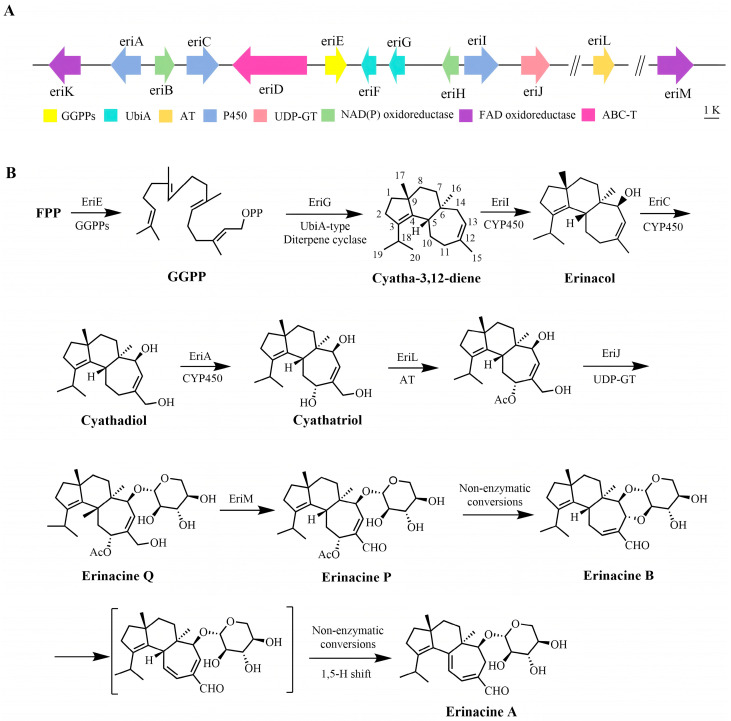
The biosynthetic pathway of erinacine A. (**A**) The organization of the *eri* gene cluster from *H. erinaceus*. (**B**) Biosynthetic pathway of erinacine A.

The rapid accumulation of fungal genomes has greatly facilitated the identification of gene clusters involved in terpenoid biosynthesis. In recent years, to fully explore the application potential of erinacines, researchers have investigated its biosynthetic pathway and associated gene clusters. Yang et al. [97] identified the erinacines synthesis gene cluster *eri* in the *H. erinaceus* genome. This cluster comprises 11 genes with the following predicted functions: EriE (GGPP synthase), EriG/F (UbiA-type diterpene cyclase), EriA/C/I (P450 enzymes), EriJ (glycosyltransferase), EriB/H (short-chain dehydrogenase), EriD (ABC transporter), and EriK (FAD-dependent oxidase) (Figure 3A). Among these, EriG is the enzyme responsible for catalyzing the cyclization of the cyathane skeleton in *H. erinaceus*, marking the first reported UbiA-type diterpene cyclase in fungi.

To further elucidate the complete biosynthetic pathway of erinacines, Liu et al. [98] performed functional validation of genes within the *eri* cluster using *Aspergillus oryzae* as the host. First, genomic DNA containing introns from EriE and G was introduced into *A. oryzae*, revealing the ability to produce cyatha-3,12-diene. This also confirms that *A. oryzae* possesses the ability to splice large fungal gene introns, making it an ideal host for studying the biosynthesis of secondary metabolites in mushrooms [99]. Based on this, three P450 genes were transferred into the *A. oryzae*, and their functions were verified as follows: EriI introduces a hydroxyl group at the C-14 position to produce erinacol; EriC is responsible for C-15 hydroxylation to produce cyathadiol; and EriA catalyzes the C-11 hydroxylation of cyathadiol to yield the compound cyathatriol. However, continuing to transfer into other genes cannot generate subsequent products. It is speculated that certain genes essential for the synthesis of moniliformin may be located outside the *eri* cluster. The absence of a key acetyltransferase gene within the cluster was noted, and the extra-cluster gene *EriL* was successfully identified via genome mining. This gene exhibits homology to *ple2*, a known acetyltransferase [99,100]. This enzyme functions by acetylating the C-11 hydroxyl group of cyathatriol, after which the glycosyltransferase EriJ utilizes UDP-xylose to glycosylate it, yielding erinacine Q. As shown in Figure 3B, Ma et al. [101] further conducted genomic mining, identifying and characterizing EriM (FAD-dependent oxidase). Using yeast, the biosynthetic pathway was reconstructed, revealing that EriM catalyzes the C-15 oxidation of erinacine Q to form the allylaldehyde intermediate erinacine P. The formation of this allylaldehyde triggers a non-enzymatic tandem Michael addition reaction, yielding the key intermediate erinacine B. Erinacine B undergoes a non-enzymatic Michael elimination reaction followed by a double bond shift, ultimately yielding the biologically active erinacine A.

## 6. Conclusions and Perspectives

Erinacine A stands out as a promising natural compound, with a growing body of preclinical evidence supporting its potent neuroprotective effects against complex pathologies like Alzheimer’s, Parkinson’s, and ischemic stroke. Its demonstrated ability to stimulate NGF synthesis, mitigate neuroinflammation, and reduce oxidative damage positions it as a strong candidate for therapeutic development. However, a significant gap remains between these compelling animal model results and proven clinical application. The majority of current findings are preclinical; therefore, future research must prioritize well-designed, large-scale human clinical trials to rigorously evaluate its safety, bioavailability, and efficacy in patient populations. The primary bottleneck impeding this clinical transition is not a lack of potential, but a challenge of supply. This review highlights that the production of erinacine A is currently confined to the mycelial fermentation of *H. erinaceus*, as the compound is absent in the fungal fruiting bodies, and its complex stereochemistry makes chemical synthesis commercially unviable. Consequently, optimizing fermentation processes—both submerged and solid-state—remains the most critical short-term strategy. Improving yields through media engineering, process parameter control, and the use of elicitors is essential for generating the quantities of erinacine A needed for advanced clinical studies.

Looking forward, the recent and successful elucidation of the complete biosynthetic pathway, including the key *eri* gene cluster and extra-cluster enzymes, opens a transformative new avenue. While optimizing the native producer is vital, the future of scalable erinacine A production likely lies in synthetic biology and metabolic engineering. By transferring the entire enzymatic cascade into a robust and industrially vetted chassis, such as Aspergillus oryzae or Saccharomyces cerevisiae, it will be possible to create high-performance “cell factories.” This heterologous expression approach, which this review’s “Biosynthesis” section shows is already feasible, would overcome the inherent limitations of the slow-growing native fungus, enabling controllable, cost-effective, and high-titer production. This biotechnological leap will be the key to unlocking the full therapeutic potential of erinacine A, moving it from a rare fungal metabolite to a widely accessible neurotrophic agent.

## Data Availability

All relevant data generated or analyzed during this study are included in this manuscript.
